# New Chalcone-Derived Molecule for the Topical Regulation of Hyperpigmentation and Skin Aging

**DOI:** 10.3390/pharmaceutics16111405

**Published:** 2024-10-31

**Authors:** Alfredo Martínez-Gutiérrez, Alexandra Bertran, Teresa Noya, Eloy Pena-Rodríguez, Susana Gómez-Escalante, Sergio Pascual, Luis Shotze Luis, Mari Carmen González

**Affiliations:** 1 Biotechnology Unit, Mesoestetic Pharma Group, 08840 Barcelona, Spain; 2 Biomedical Engineering Unit, Mesoestetic Pharma Group, 08840 Barcelona, Spain; 3 Medical Affairs, Mesoestetic Pharma Group, 08840 Barcelona, Spain; 4 R+D Department, Mesoestetic Pharma Group, 08840 Barcelona, Spain

**Keywords:** pigmentation, epigenetics, skin aging, chemical synthesis, cell culture

## Abstract

**Background/Objectives**: Skin hyperpigmentation is a biological process that results in an excessive production of melanin and is highly regulated by several mechanisms, tyrosinase being one of the key enzymes involved. Current reported inhibitors lack clinical efficacy, show toxic side effects, have poor bioavailability, or low formulation compatibility. The aim of this study was to design a new effective tyrosinase inhibitor for topical hyperpigmentation and anti-aging treatments. **Methods**: Homology modeling was used to build the tridimensional structure of human tyrosinase, and virtual docking was used to predict molecule–enzyme binding modes. The tyrosinase activity of the designed and synthesized compounds was assessed and water solubility was determined by HPLC. Cell assays were performed to determine melanin content, cytotoxicity, wound healing, anti-glycation, antioxidation, and autophagy efficacy. Gene expression and miRNA levels were quantified by qPCR and chromatin accessibility by ATAC-Seq. Human reconstructed epidermis was used to test the depigmenting efficacy as well as the skin irritation potential. **Results**: The 3D structure of human tyrosinase was designed and validated. The new molecule could effectively inhibit human tyrosinase and melanin synthesis in 2D monocultures and a 3D epidermis model. Two melanogenesis-related miRNAs were increased in treated cells. Anti-glycation, antioxidant, mitochondria protection, autophagy activation, and wound healing properties were also observed, with special emphasis on epigenetics. **Conclusions**: The designed molecule is a potential candidate to be used as a depigmenting and anti-aging agent, with suitable properties to be introduced in final product formulations for dermatology or cosmetics treatments.

## 1. Introduction

Melanogenesis is a process driven by the synthesis of melanin, which is produced by the enzymatic oxidation of L-tyrosine in melanocytes [1]. Although it plays a physiological role protecting skin from UV damage, excessive accumulation of this pigment occurs in some skin conditions such as melasma, post-inflammatory hyperpigmentation, or solar lentigo [2].

Tyrosinase is a copper-containing enzyme demonstrated to be a key regulator in the process of melanogenesis. Thus, the inhibition of this enzyme has been selected as the main approach to treat skin hyperpigmentation [3,4]. However, among the numerous tyrosinase inhibitors that have been reported in the scientific literature, the majority show at least one drawback (lack of clinical efficacy, toxic side effects, poor bioavailability, or chemical instability) that limits their use in topical formulations [4,5]. Among the described chemical families targeting this enzyme, chalcone derivatives have promising results showing a potent inhibitory activity against tyrosinase [6,7]. These chemical entities have advantages over other families of compounds, as they show promising biological activities such as antioxidant, antimicrobial, anti-aging, anti-inflammatory, or antiviral activities, among others [8,9,10]. Moreover, these compounds are also easy to modify and synthesize by basic chemistry reactions. In fact, a wide range of derivatives can be synthesized due to the large number of substitutable hydrogen atoms. These features make chalcones promising scaffolds for the discovery of new chemical entities [8,9,10]. One example of a designed chalcone-based molecule is a phloretin sulfonated derivative that shows good water solubility properties while exerting anti-photoaging effects on skin [11].

Thus, despite the clear advantages of chalcones, these kinds of chemical entities have limitations as topical ingredients such as low solubility, showing values of water solubility lower than 1 µg/mL [8,12,13]. For this reason, there is a need for finding new chemical entities that have both inhibitory activity for tyrosinase and increased water solubility, key parameters for their introduction in skin depigmentation formulations. Increased water solubility allows for the incorporation of water-soluble compounds in topical galenic emulsions formed by both an aqueous and oily phase.

Apart from pigmentation disorders, another feature of UV damage is the rise of skin aging processes such as the formation of wrinkles, loss of elasticity, or roughness. Several molecular mechanisms have been described as protective against the aging process, such as autophagy [14], a reduction in advanced glycation end products (AGEs) [15], or antioxidation [16], as some examples.

The aim of this study was to develop a new chalcone-related molecule that is effective against the hyperpigmentation process while being a good candidate to be included in topical formulations. The design involved predicting the 3D structure of human tyrosinase used to design chemical entities that both inhibit the enzyme activity in an efficient manner and have higher solubility in aqueous-based formulations. Chalcones have been described as modulators of age-related processes [17]; therefore, the anti-aging properties of the new molecule were also investigated.

## 2. Materials and Methods

### 2.1. Computational Design

The structural model of human tyrosinase was built using the MAFFT tool [18] by the alignment of sequences between tyrosinase-related (PDB: 5M8P and 5M8S) and human tyrosinase (EC 1.14.18.1). Then, the obtained structure was aligned with TYRP1 structures (5M8S and 4P6R), and the resulting coordinates were extracted and incorporated manually by a homology model (MODELER 9.1, Hitachi Zosen Information Systems Co., Ltd., Osaka, Japan) [19]. The resulting tridimensional structure was compatible with the ionic configuration of the tyrosinase activity [20].

The 3D structure was validated by interacting the tyrosinase active site and its natural substrate, L-tyrosine. The binding modes between tyrosinase and the molecules were predicted by virtual docking (AutoDock 4.2, The Scripps Research Institute, La Jolla, CA, USA) [21].

### 2.2. Synthesis of Compounds

#### 2.2.1. Synthesis of 5-Acetyl-2-hydroxybenzenesulfonic Acid (Intermediate)

An amount of 2 g (0.015 mol) of 1-(*p*-hydroxyphenyl)-1-ethanone was added dropwise to 17.6 g (0.151 mol) of chlorosulfonic acid at 0 °C. After 18 h at RT, 75 mL of deionized water was added, and the mixture was extracted with ethyl acetate. The organic phase was evaporated until dryness. A total of 1.71 g (0.008 mol) of crude product containing 5-acetyl-2-hydroxybenzenesulfonic acid was obtained (yield 54%).

^1^H NMR (d-DMSO, 360 MHz): δ 2.50 (3H), 6.88 (d, 1H), 7.84 (d, 1H), 8.06 (s, 1H).

#### 2.2.2. Synthesis of 5-[(*E*)-3-(*p*-Hydroxyphenyl)acryloyl]-2-hydroxybenzenesulfonic Acid (Compound **1**)

A volume of 1.61 mL of sulfuric acid 98% was added dropwise to a suspension of 563 mg (0.003 mol) of the obtained crude (intermediate) product and 362 mg (0.003 mol) of p-hydroxybenzaldehyde in ethanol at 0 °C. The reaction was stirred for 21 h at RT and the obtained suspension was added dropwise to a solution of 0.2 g/mL of sodium chloride in water at 0 °C. The suspension was extracted with ethyl acetate and the organic phase was evaporated until dryness. A total of 230 mg (0.001 mol) of crude product containing 5-[(E)-3-(*p*-hydroxyphenyl)acryl]-2-hydroxybenzenesulfonic acid was obtained (yield 28%).

^1^H NMR (d-DMSO, 360 MHz): δ 6.83 (d, 2H), 6.94 (d, 1H), 7.66 (d, 2H), 7.72 (d, 2H), 8.11 (d, 1H), 8.23 (s, 1H).

IR spectrum: 1227 cm^−1^ (s, R−S(=O)_2_−OH, sulfonic group), 1505 (s, C=C, aromatic), 1524 (m, C=C, aromatic), 1549 (s, C=C, aromatic), 1585 (m, C=C, aromatic) (where s: strong; m: medium). The spectrum is shown in the Supplementary Information.

#### 2.2.3. Synthesis of 5-[(*E*)-2-(4-Hydroxy-3-sulfobenzoyl)-1-ethenyl]-2-hydroxybenzenesulfonic Acid (Compound **2**)

An amount of 1.04 g (0.004 mol) of (*E*)-1,3-bis(*p*-hydroxyphenyl)-2-propen-1-one was added dropwise to 5.04 g (0.043 mol) of chlorosulfonic acid at 0 °C. The suspension was stirred at RT for 22 h, and then 25 mL of water was added to the obtained mixture. The aqueous phase was extracted with ethyl acetate and the organic phase was evaporated until dryness. A total of 1.49 g (0.003 mol) of crude product containing 5-[(*E*)-2-(4-hydroxy-3-sulfobenzoyl)-1-ethenyl]-2-hydroxybenzenesulfonic acid was obtained (yield 86%).

^1^H NMR (d-DMSO, 360 MHz): δ 6.86 (d, 1H), 6.91 (d, 1H), 7.62 (d, 1H), 7.70 (d, 1H), 7.82 (d, 1H), 7.86 (sd, 1H), 8.14 (s, 1H), 8.21 (s, 1H).

#### 2.2.4. Synthesis of (*E*)-1,3-Bis(*p*-hydroxyphenyl)-2-propen-1-one (Compound **3**)

An amount of 16.06 g of sulfuric acid (98%) was added dropwise to a mixture of 2 g (0.016 mol) of *p*-hydroxybenzaldehyde and 2.23 g (0.016 mol) of 1-(*p*-hydroxyphenyl)-1-ethanone. The reaction was stirred for 23 h at RT and the obtained mixture was added dropwise to a solution of 10 g of NaCl and 33 mL of water at 0 °C. The aqueous phase was extracted with ethyl acetate and the organic phase was evaporated until dryness. A total of 3.58 g (0.015 mol) of crude product containing (*E*)-1,3-bis(*p*-hydroxyphenyl)-2-propen-1-one was obtained (yield 91%).

^1^H NMR (d-DMSO, 360 MHz): δ 6.83 (d, 2H), 6.88 (d, 2H), 7.63 (d, 2H), 7.69 (d, 2H), 8.02 (d, 2H).

### 2.3. Solubility Assay

Compounds were dissolved at a final concentration of 50 mg/mL in DMSO and in water. The obtained solutions were stirred for 1 h at room temperature (RT), then filtered and analyzed by HPLC. The solubility percentage of each compound was calculated by taking the peak area obtained by HPLC from the sample dissolved in water divided by the compound in DMSO.

### 2.4. HPLC Conditions

The HPLC system consisted of an Agilent 1260 Infinity II Prime. The HPLC column used was Phenomenex™ LUNA C18 100 Å (250 mm × 4.6 mm × 5 µm) (Phenomenex, Torrance, CA, USA). The chromatographic method was as follows: 90% mobile phase A (1.3 mg/mL (NH_4_)_2_HPO_4_ at pH 7.2) and 10% mobile phase B (acetonitrile). Then, a linear gradient elution up to 60% acetonitrile in 30 min was performed. Flow rate: 1.0 mL/min; injection volume: 3 µL; column temperature: 25 °C.

### 2.5. Cell Cultures

Human primary adult melanocytes (HEMs) (Cellsystems GmbH, Troisdorf, Germany) were cultured in DermaLife™ Ma medium supplemented with Zellshield (Minerva Biolabs GmbH, Berlin, Germany). Human dermal fibroblasts (HDFs) (Promocell, Heidelberg, Germany) were cultured in DMEM (Capricorn Scientific GmbH, Ebsdorfergrund, Germany) supplemented with 10% fetal bovine serum (Fisher Scientific, Hampton, NH, USA) and Zellshield (Minerva Biolabs GmbH). Both cell types were incubated at 37 °C in 5% CO_2_ and 90% humidity.

### 2.6. Cell Viability Assay

The cell viability assay of compound **1** against HEMs and HDFs was measured using the SRB assay, as previously reported elsewhere [22]. Briefly, cells were incubated for 24 h and then treated with different concentrations (3.125–1562.5 μM) of compound **1** for 72 h. Then, 10% trichloroacetic acid was added to the cells and incubated at 4 °C for 1 h. The SRB solution was added on cells that were incubated for 30 min at RT. The SRB solution was removed, and cells were washed 4 times with 1% acetic acid, and they were allowed to dry at RT. The dye was dissolved with 10 mM Tris base solution (pH 10), and the absorbance was measured at 540 nm using a microplate reader.

### 2.7. ATAC-Seq Study

HDFs were treated for 24 h with compound **1** at 312.5 µM. After treatment, treated and non-treated cells were harvested, pelleted, and frozen. The cells were then thawed in a 37 °C water bath, pelleted, washed with cold PBS, and tagmented, as previously reported elsewhere [23], with some modifications based on a previous report [24]. An analysis of ATAC-seq data was performed, as previously reported elsewhere [25].

### 2.8. Gene Expression and miRNA Level Quantification by qPCR 

RNA was extracted from cell pellets using the Total RNA Purification Kit (Norgen, Thorold, ON, Canada) according to the manufacturer’s protocol. Extracted RNA was quantified using a NanoDrop™ ND-1000 UV–Vis spectrophotometer (Thermo Fisher Scientific, Waltham, MA, USA).

For gene expression quantification, 300 ng of RNA was retrotranscribed to cDNA using the PrimeScript™ RT reagent kit (Takara Bio Inc., Shiga, Japan). cDNA was diluted 1/10 in ultrapure water and the qPCR mix was prepared by adding cDNA (12 ng), SYBR Green Supermix (Bio Rad, Hercules, CA, USA), and forward and reverse primers (0.2 μM). The primers and their respective sequences are listed in Supplementary Tables S1 and S2. The qPCR protocol included one step at 95 °C for 30 s, 40 cycles at 95 °C for 10 s, and 60 °C for 1 min. The results were analyzed with Bio-Rad™ CFX Manager software (version 3.1). ACTB was used as a housekeeping gene to normalize gene expression.

For miRNA level quantification, Mir-XTM miRNA First Strand Synthesis kit (Takara Bio Inc., Japan) was used to generate cDNA from miRNAs. This kit contains the universal 3′ primer and the 5′ and 3′ primer for U6 to be used as a housekeeping gene. The 5′ primer for each miRNA corresponds to its sequence (Supplementary Table S3). The qPCR protocol included one step at 95 °C for 10 s, 40 cycles at 95 °C for 5 s, and 60 °C for 20 s.

### 2.9. Tyrosinase Inhibition Assay

HEMs were incubated for 72 h with L-tyrosine at 2 mM. Tyrosinase extraction and an enzyme assay were performed following the protocol of Winder and Harris [26]. Briefly, cells were harvested and lysed in 100 µL PBS at pH 7 containing 1% Triton X-100 during 30 min at 4 °C. Lysates were then centrifuged at 14,000× *g* for 15 min. Finally, 90 µL of supernatants containing tyrosinase were incubated in 96-well plates with 6 µL MBTH 126 mM, 10 µL L-DOPA 12.6 mM, and 20 µL of PBS or the compound to be tested. Absorbance at 492 nm was quantified after 60 min of reaction using Multiskan™ FC Microplate Reader (Thermo Fisher Scientific, USA).

### 2.10. Melanin Inhibition Assay

Melanin content was measured, as previously reported elsewhere [27], with minor modifications. After 24 h of incubation, HEMs were treated with an aqueous solution consisting of 2 mM L-tyrosine and 312.5 µM compound **1** and incubated for 72 h. After treatment, cells were trypsinized and cell pellets were dissolved in 1 M NaOH at 80 °C for 30 min. The absorbance was measured at 340 nm using Multiskan™ FC Microplate Reader (Thermo Fisher Scientific, USA).

### 2.11. Anti-Glycation Effect

Bovine serum albumin and glyceraldehyde glycation product (BSA-GYA) were used to induce damage caused by advanced glycation end products (AGEs). HDFs were incubated with 1 mg/mL BSA plus 0.4 mg/mL GYA (with or without compound **1**) at 312.5 µM for 24 h. Cell viability was determined by SRB quantification, as described in the previous section. For the live-cell imaging assay, cells were treated in the same conditions as before for 24 h. Cells were recorded using the 3D Cell Explorer™ microscope (Nanolive, Tolochenaz, Switzerland). An image of the last hour of incubation was then extracted to compare the morphology of the cells after the different treatments.

### 2.12. Antioxidant Activity Assay

For the antioxidant assay, HDFs were treated with menadione (10 μM), compound **1** (312.5 µM), and CellROX™ (5 µM) for 6 h in the incubator. For the mitochondria protection assay, HDFs were treated with menadione and compound **1** for 24 h and then incubated with Mitotracker™ (200 nM) for 30 min in the incubator. In both cases, cells were washed with PBS and fluorescent pictures using the FITC channel (excitation 474 nm/emission 515 nm) were taken using the 3D Cell Explorer™ microscope.

### 2.13. Wound Healing Assay

The wound healing assay was performed, as previously reported elsewhere [28], with some modifications. HDFs were seeded and after 24 h of incubation, a linear scratch was generated using a pipette tip. Then, compound **1** was added with a starved cell medium (fetal bovine serum 0.5%) for 24 h and cells were imaged with a CKX41 phase contrast microscope (Olympus, Tokyo, Japan). Regenerative capacity was quantified by counting the number of cells in the region of interest (ROI) on the scratch. This quantification was performed in 3 representative pictures for each condition using the Cell Counter plugin of ImageJ software (ImageJ2 v2.35, National Institutes of Health, Bethesda, MD, USA).

### 2.14. RHPE Depigmentation Efficacy Assay

The RHPE of phototype VI, at an age of 10 days and with a 0.5 cm diameter were used (Episkin, Lyon, France) according to the manufacturer’s instructions. Upon receipt of the tissues, they were incubated overnight at 37 °C in 5% CO_2_ with fresh Episkin Growth Medium™ (Episkin, Lyon, France). Tissues were then treated with PBS (control) and a 0.5% *w*/*w* compound **1** solution in PBS. A 25 µL treatment of both formulations was applied after 1, 2, 5, 6, and 7 days. Washes with PBS were performed after each treatment, and the medium was replaced with fresh medium every 24 h.

Twenty-four hours after the last treatment, cell viability assays were performed by MTT with duplicates of each formulation according to the manufacturer’s instructions [29].

Tissue melanin was semi-quantitatively analyzed by immunohistochemistry using a Fontana–Masson staining kit™ according to the manufacturer’s instructions (Abcam, Cambridge, UK). Tissues were fixed with 4% *w*/*w* paraformaldehyde (PFA) at 4 °C. Transverse sections 10 μm thick were obtained by paraffin embedding at 4 °C and microtomy. 

Representative images were obtained by optical microscopy on a Leica DM6000 microscope (Leica Biosystems, Nussloch, Germany) at a 20× magnification. A semiquantitative 2D melanin analysis was performed using ImageJ software (ImageJ2 v2.35, National Institutes of Health, Bethesda, MD, USA). The black stained pixels (melanin) were compared semi-quantitatively between the two formulations.

### 2.15. Skin Irritation Test

Skin irritation was assessed using RHE from MatTek corporation (EPI-200-SIT) according to the OECD 439 test method and following the validated EpiDerm™ Skin Irritation Test.

### 2.16. Statistical Analysis

The experiments were performed three times under the same conditions, and the average and statistical significance were determined with Student’s *t*-test (* *p* < 0.05, ** *p* < 0.01, *** *p* < 0.001) using Microsoft Excel™ version 2408 (Microsoft Corporation, Redmond, WA, USA).

## 3. Results

### 3.1. Computational Approach for the Identification of Novel Tyrosinase Inhibitors and Chemical Synthesis

The binding affinity between tyrosinase and its natural substrate, L-tyrosine, was predicted by virtual docking showing an affinity of −4.8 kcal/mol. This result validated the designed model of human tyrosinase; thus, it is suitable to predict binding affinities with other compounds.

**Figure 1:**
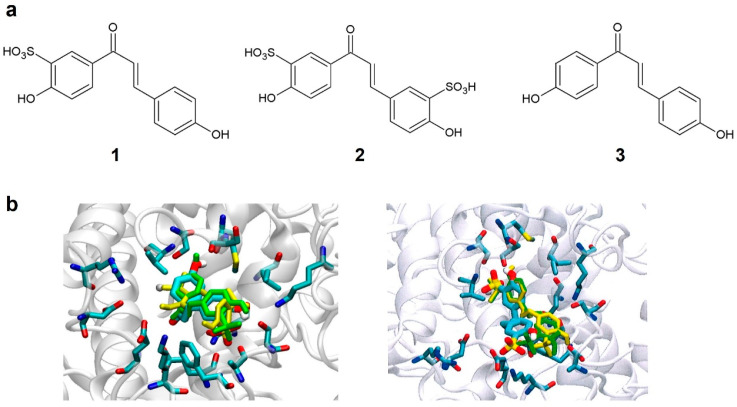
(**a**) Structure of designed tyrosinase inhibitors (**1** and **2**) and the reference compound **3**. (**b**) Binding mode of compounds **1** (**left**) and **2** (**right**) with human tyrosinase.

Compounds **1** and **2** (Figure 1), designed by the sulfonation of the chalcone structure at the ortho position of hydroxyl groups, were selected for the computational analysis because of their increased chances of solubility in water due to the presence of the sulfonate group and chemical synthesis feasibility. Both compounds were subjected to docking studies using the human tyrosinase model. Results indicated that the binding mode simulation of the proposed structures was improved compared to the natural substrate, with affinities of −8.0 and −8.1 kcal/mol for compounds **1** and **2**, respectively. Both compounds showed a similar degree of interaction. The docking analysis revealed interactions of compound **1** with Met374 and Lys334 of tyrosinase. A similar degree of binding was found for compound **2** interacting with residues Ser380, Met374, Asn364, Lys334, Phe347, and Ala357. These results suggest that the sulfonic acid group in the ortho position is crucial to increase interactions between compounds **1** and **2** and the tyrosinase active site.

Compounds **1** and **2**, as well as the reference compound, were then synthesized according to the procedure described in the Section 2. The synthetic schemes of the three compounds are shown in Supplementary Figure S1.

### 3.2. Solubility Study

HPLC was used to compare the aqueous solubility of synthesized molecules **1** and **2** with the reference chalcone **3** (Figure 1a). The reference compound showed no solubility in water at 50 mg/mL. On the contrary, compounds **1** and **2** were almost completely dissolved in water, showing solubilities of 84% (42 mg/mL) and 81% (40.5 mg/mL), respectively.

### 3.3. Tyrosinase Activity

Tyrosinase inhibition in cell extracts from melanocytes treated with compounds **1**, **2**, and **3** was 56%, 22%, and 16%, respectively. The positive control compounds, hydroquinone and kojic acid, showed a mild inhibition of 16% and 23%, respectively (Table 1).

### 3.4. Cell Viability

Compound **1** was found to have no effect on HEM and HDF viability at concentrations lower than 312.5 µM. At higher concentrations (≥1562.5 µM), this compound showed significant cytotoxicity with respect to the control without treatment (Table 2). The maxi-mum non-cytotoxic dose of 312.5 µM was used in the subsequent cellular assays to determine the efficacy and mechanism of action of compound **1**.

### 3.5. Melanin Inhibition

**Figure 2:**
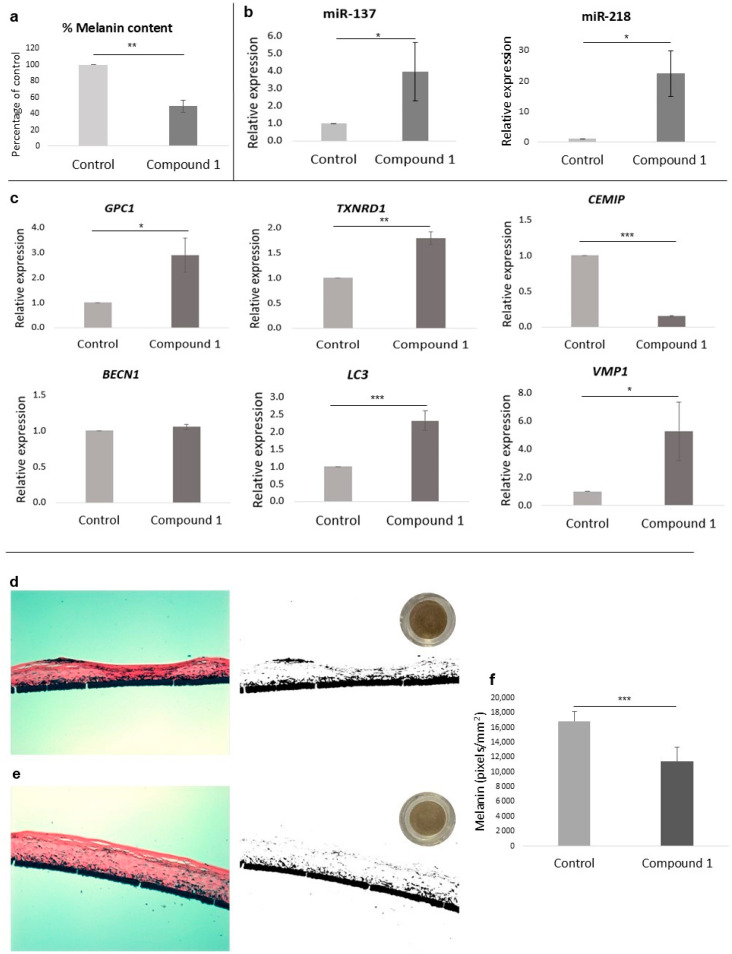
(**a**) Percentage of melanin content of melanocytes treated with compound **1** at 312.5 µM. (**b**) qPCR analysis of miR-137 and miR-218 levels in melanocytes treated with compound **1** at 312.5 µM. (**c**) qPCR analysis of *GPC1*, *TXNRD1*, *CEMIP*, *BECN1*, *LC3*, and *VMP1* gene levels in fibroblasts treated with compound **1** at 312.5 µM. (**d**,**e**) Histological cross-sections and melanin semi-quantification by Fontana–Masson image analysis of reconstructed human pigmented epidermis (phototype VI) treated for 8 days. (**d**) Model treated with PBS (control) and (**e**) model treated with 0.5% *w*/*w* compound **1**. Representative images at 20× magnification (left), binary black and white mask (right), and bottom-up scanned tissue (upper right). (**f**) Two-dimensional melanin normalized semi-quantification results (pixels/mm^2^, *n* = 8). Bar graphs indicate mean ± SD (*n* = 3). * *p* < 0.05; ** *p* < 0.01; *** *p* < 0.001 (Student’s *t*-test).

Melanin accumulation in melanocytes treated for 72 h with L-tyrosine and compound **1** was reduced to 49% compared to melanocytes treated with L-tyrosine alone (Figure 2a).

### 3.6. Epigenetic Effect on Depigmentation

Among the tested miRNAs involved in melanogenesis regulation, levels of miR-137 and miR-218 were significantly increased (*p* < 0.05) after the treatment with compound **1** compared to the control group (Figure 2b).

### 3.7. Effects on Chromatin Accessibility

The chromatin accessibility study indicated that there were three genes (*CEMIP*, *PRPF6*, and *VEPH1*) downregulated and six genes (*TXNRD1*, *FTH1*, *BECN1*, *TDP2*, *GPC1*, and *FN1*) upregulated in cells treated with compound **1** for 24 h compared to non-treated cells.

### 3.8. Effects on Anti-Aging Gene Expression

To further validate the results obtained in the ATAC-Seq study, qPCR was performed using specific primers of the previous anti-aging genes. After incubating HDFs with compound **1** for 24 h, the genes *GPC1* and *TXNRD1* were significantly increased compared with the untreated group. On the contrary, *CEMIP* was downregulated. As the *BECN1* gene did not change after the treatment, we quantified other interesting genes involved in autophagy. Among these, *LC3* and *VMP1* were upregulated by compound **1** treatment (Figure 2c).

### 3.9. Anti-Glycation Activity 

**Figure 3:**
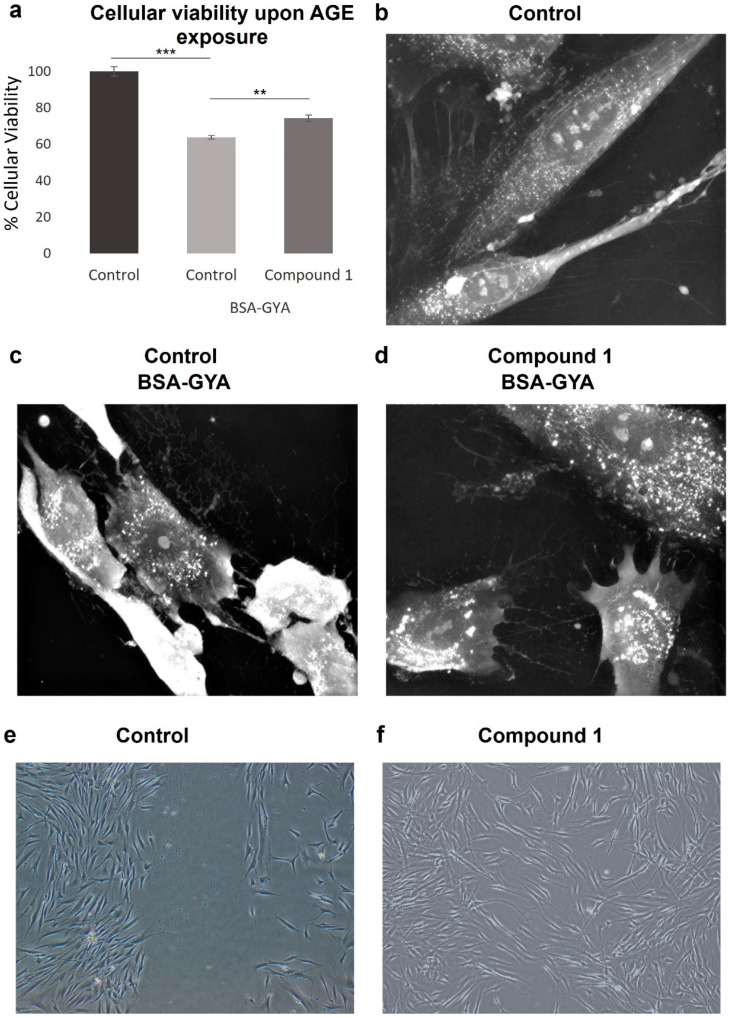
Anti-glycation and wound healing effects of compound **1**. (**a**) Cellular viability of non-treated and BSA-GYA- and compound **1**-treated human dermal fibroblasts (HDFs). Time-lapse imaging of HDFs after 24 h of (**b**) no treatment, (**c**) BSA-GYA treatment, and (**d**) compound **1** and BSA-GYA treatment (60× magnification). (**e**,**f**) Representative images from wound healing assay of non-treated and compound **1**-treated HDF (5× magnification). Bar graphs indicate mean ± SD (*n* = 3). ** *p* < 0.01; *** *p* < 0.001 (Student’s *t*-test).

The SRB assay of fibroblasts treated with BSA-GYA plus compound **1** for 24 h showed that the compound was able to partially protect against the cytotoxic effects induced by BSA-GYA compared to the control stimulated without compound **1** (74.3% vs. 63.7% cell viability, Figure 3a).

The effects of compound **1** on glycation were also investigated by live-cell imaging. BSA-GYA treatment induced cell death after 6 h of incubation, as appreciated in the change in morphology towards cell shrinkage and membrane blebbing (Figure 3c). When compound **1** was added with the glycation inducers, morphologic changes occurred to a lower extent and cell death was not observed (Figure 3d), meaning that this molecule is protecting from cell damage induced by glycation. Interestingly, an increase in intracellular white dots is also observed with the treatment with BSA-GYA plus compound **1**. This could correlate with changes in intracellular organelles such as endocytic vesicles or lysosomes as part of the stress response induced to protect cell integrity.

### 3.10. Wound Healing Effect

Microscope images of fibroblasts treated for 24 h with compound **1** after scratch show that 112 ± 22 cells migrated into the scratch in compound **1**-treated cells compared to 32 ± 14 cells in non-treated cells, indicating a significant regeneration response induced by compound **1** (** *p* < 0.01) (Figure 3e,f).

### 3.11. Antioxidant Activity

**Figure 4:**
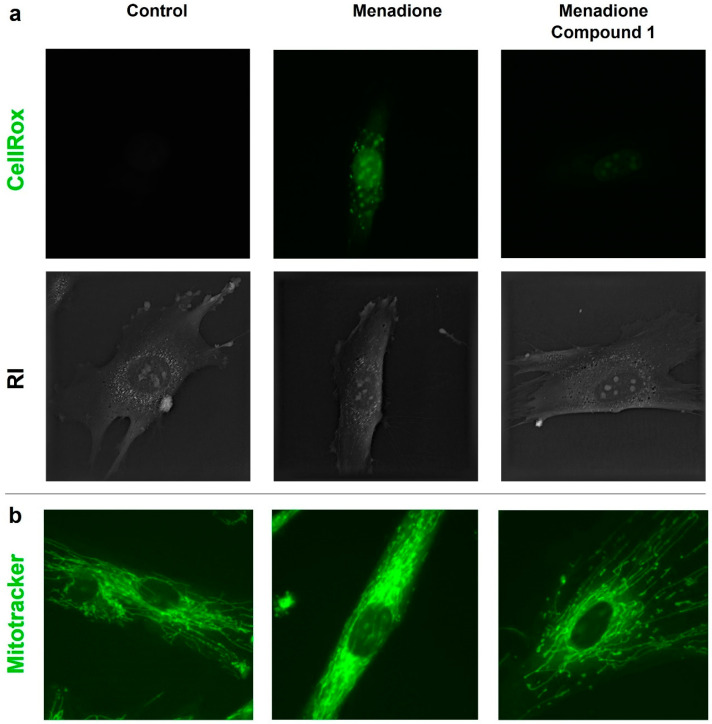
(**a**) Representative comparison of CellROX™ fluorescent signals and refractive index (RI) distribution for non-treated, menadione-, and menadione + compound **1**-treated HDFs (60× magnification). (**b**) Representative fluorescent images of Mitotracker™ signal for non-treated, menadione-, and menadione + compound **1**-treated HDFs (60× magnification).

The live-cell imaging assay using the fluorescent dye CellROX™ showed that menadione itself increased the fluorescence intensity after 24 h of incubation. On the contrary, the co-treatment of menadione with compound **1** resulted in a significant reduction in fluorescence intensity, as observed with the non-treated HDF, meaning that the compound can neutralize the generated reactive oxygen species (ROS) generated by menadione (Figure 4).

A similar experiment with the live-cell microscope was performed using HDFs treated with menadione and compound **1** (312.5 µM) after 24 h of treatment. As shown in Figure 4b, the fluorescence signal suggested that menadione itself changed the mitochondrial morphology. When compound **1** was co-treated with menadione, the mitochondrial morphology was similar to the non-treated HDFs.

### 3.12. Skin Irritation

The MTT assay performed according to OECD 439 indicated that the cell viability of the reconstructed human epidermis (RHE) treated with compound **1** was 83%, suggesting a non-irritant potential of the compound.

### 3.13. Reconstructed Human Pigmented Epidermis (RHPE) Depigmentation Efficacy

A visual comparison of the tissues from above (circular tissue images in Figure 2d,e) showed the depigmenting effect of compound **1**. When analyzing the histological sections (Figure 2d,e; left images), no signs of cytotoxicity were observed. Furthermore, when looking at the binary masks (Figure 2d,e; black and white images), a reduction in melanin accumulations (black) is observed, especially in the more superficial layers of the epidermis. After 2D semi-quantification, it was possible to observe significant differences after analyzing eight different histological slices for each treatment (Supplementary Figures S3 and S4), with a significant reduction (*** *p* < 0.001) of approximately 33% in melanin compared to the untreated control.

## 4. Discussion

Chalcone derivatives are promising molecules with high tyrosinase inhibitory activity, a key enzyme involved in melanogenesis. However, these molecules have a low solubility and high-toxicity profile; thus, their use in final product formulations is limited [8]. The main objective of this study was to develop a new chalcone-based compound that could be used effectively as a novel depigmenting agent. The design was based on the identification of a chalcone-based molecule able to bind the active center of tyrosinase and inhibit its function.

It has been proven that the sulfonation process to substitute a hydrogen atom with sulfonic acid can increase the water solubility of molecules and is suitable from the syn-thetic chemistry point of view [30]. Starting from the chalcone substructure and hydroxyl groups at key positions of the phenyl rings [5], two molecules (compounds **1** and **2**) were proposed based on the mono- and disulfonation of the chalcone derivative (compound **3**) at the ortho position of the hydroxyl groups.

A computational design was performed to validate the suggested molecules as potential tyrosinase inhibitors. We developed a new model of tyrosinase that reproduced not only its structure but also the ionic distribution compatible with the redox activity of endogenous human tyrosinase [20,31]. Based on these requirements, a new 3D model was designed and then validated successfully by L-tyrosine, the native substrate of tyrosinase. The two proposed chalcone-based compounds (**1** and **2**) showed increased binding affinities compared to the native substrate, thus validating both as potential tyrosinase inhibitors. Hence, the sulfonation modification was suitable based on its ability to increase water solubility, its increased binding affinity of the modified compounds towards the active site, and its feasibility regarding the synthetic preparation.

The designed compounds were first synthesized and then evaluated experimentally. The in vitro tyrosinase inhibition of both compounds was tested using HEMs stimulated with L-tyrosine. This substrate is a key component involved in the synthesis of melanin and can activate cellular receptors that trigger signaling pathways involved in the melanogenesis process [32]. The melanocyte lysates obtained contained mainly tyrosinase, among other melanogenesis-related enzymes. For this reason, tyrosinase inhibition values obtained in this work were not only associated with tyrosinase inhibition but also to other related enzymes, indicating that their efficacy resembled more what occurs in intact skin. Results indicated that compound **1** was a stronger tyrosinase inhibitor than hydroquinone and kojic acid, while compound **2** showed similar results to the reference compounds. Furthermore, both chalcones showed an outstanding increase in water solubility compared to the chalcone-based standard. Interestingly, the tyrosinase inhibition of compounds **1** and **2** is significantly different (56% vs. 22%) despite the chemical similarity. This difference might be explained by the fact that the extra sulfonate group in compound **2** is interacting with amino acids that are located outside the active site. Compound **1**, showing higher tyrosinase inhibition, was finally selected as the candidate with the best depigmenting potential, considering that it also reduced melanin synthesis.

Chalcones have been described in the literature as possessing a wide range of biological activities aiming to revert age-related processes such as oxidative stress, glycation, or autophagy, among others [17,33]. The anti-aging properties of the new molecule were studied to elucidate the mechanism of action of the selected compound in cellular assays, with a special focus on the gene expression profile. To achieve this, HEMs were used to study depigmentation mechanisms and HDFs for anti-aging processes.

First, we studied the effect of compound **1** in epigenetic changes associated with depigmentation. Results indicated that the levels of two miRNAs (miR-137 and miR-218) were increased in its presence. Both epigenetic regulators are described to downregulate the gene expression of MITF, a key transcription factor involved in the melanogenesis process [34].

Then, an ATAC-Seq study with compound **1** was performed to identify the accessibility of chromatin regions and, consequently, the regulation of gene expression. The genomic profiling resulted in a total of nine alterations in chromatin regions relevant to skin processes. The related genes were found to be regulators of anti-aging processes. We observed a decreased chromatin opening of *VEPH1*, which is involved in the regulation of TGF-β signaling [35], and *CEMIP*, involved in the metabolism of hyaluronic acid [36,37]. Genes with increased chromatin opening were *BECN1*, involved in the signaling pathway of autophagy [38], *GPC1* and *FN1*, involved in wound healing [39], *TXNRD1* and *FTH1*, involved in redox processes [40,41,42,43], and *TDP2*, involved in DNA repair [44].

It has been reported that the transcriptional activity of a gene is controlled by the conformational distribution of chromatin subunits [45,46]. Here, we correlated the changes in the chromatin regions with the expression levels of related genes by qPCR. Some genes such as *GPC1*, *TXNRD1*, and *CEMIP* were altered, thus proving the effect predicted by the ATAC-Seq study. Although *BECN1* was not altered by qPCR, other genes involved in the autophagy process, such as *LC3* and *VMP1*, were induced by compound **1** treatment.

Ultimately, we aimed to correlate the changes observed in gene expression with functional cell assays including oxidation, glycation, and wound healing mechanisms. For this, live-cell imaging in combination with other in vitro techniques were used to further elucidate the mechanism of action of compound **1**.

Menadione is a widely used pro-oxidant agent, inducing oxidative stress, which progressively increases the intracellular concentration of ROS [47]. The effects of menadione were observed using live-cell imaging with two fluorescent dyes. On one hand, Mitotracker™ was used to label mitochondrial structural changes caused by the oxidative damage. On the other hand, the oxidative stress induced by menadione was detected from labeling ROS generated by CellROX™. We observed that compound **1** was able to revert the oxidative response caused by menadione, thus demonstrating its antioxidant effect.

Advanced glycation end products are the result of chemical reactions between skin proteins (e.g., collagen or elastin) and carbohydrates (e.g., glucose). During the aging process, AGEs accumulate in the skin and lead to changes in the structure of proteins, causing a loss of function. Glycation may also cause subsequent oxidative damage, inflammation, and cellular damage, leading to premature skin aging and losing skin firmness [48,49]. In this study, AGEs were produced by the reaction between BSA and GYA, exerting cytotoxicity on HDFs. Two experiments (SRB assay and live-cell imaging) were performed to prove that compound **1** protected effectively against the cellular damage caused by AGEs. Therefore, this new compound can be considered a promising candidate for the inhibition of glycation-mediated aging.

The implication of compound **1** in the process of wound healing was suggested by epigenetic studies (*GPC1* and *FN1* genes). To corroborate these effects, a functional assay to evaluate wound regeneration was performed in vitro, and results suggested an effective recovery of a scratched wound by cells treated with compound **1**. These results validated the use of compound **1** in regeneration processes and skin rejuvenation.

When using in vitro 2D models of melanocyte monocultures, the skin barrier function is not considered. To study depigmentation in a model closer to human skin, 3D skin models with RHPE were used. No signs of cytotoxicity and a significant depigmentation of tissues were observed after treating the RHPE model with 0.5% *w*/*w* compound **1** for 8 days. Although the RHPE model approximates human skin and its lipid barrier is somewhat lower than that of real human skin, these results indicate good bioavailability in human tissue for compound **1** after topical application, a property that several tyrosinase inhibitors are lacking [4,5]. However, cytokine production after compound exposure and bioavailability in vivo should be tested to confirm the skin penetration and non-irritant properties of the compound.

In this research, we have proven that the new chalcone-based molecule protects HDFs from glycation-induced cell death. Nevertheless, further investigation may be performed to understand the specific mechanisms by which the chalcone derivative molecule protects cell damage at the molecular level. It will also be interesting to discover what other proteins are regulated by the molecule to explore new therapeutic approaches and understand this novel molecule in more detail.

## 5. Conclusions

The computational design, synthesis, and evaluation of a new tyrosinase inhibitor is presented in this research. Its mechanism of action has been elucidated at the gene and protein level, with special emphasis on the involvement in epigenetic mechanisms. Interestingly, this compound is not only an effective tyrosinase inhibitor but also induces the levels of miRNAs involved in melanogenesis regulation and possesses anti-aging properties such as antioxidant, anti-glycation, autophagy, and wound healing effects. In addition, compound **1** has been shown to be effective in 3D models of RHPE at non-irritant doses. These results lead us to conclude that the newly designed molecule is a promising candidate to be used as an effective depigmenting and anti-aging agent with suitable properties to be introduced in dermatology and cosmetics formulations.

## Figures and Tables

**Table 1:** Tyrosinase inhibition percentage of compounds **1** and **2** and the reference **3**, hydroquinone and kojic acid, at 1 mM. Results indicate mean ± SD (*n* = 3).

Treatment	Tyrosinase Inhibition
Control	0 ± 2%
Compound **1**	56 ± 1%
Compound **2**	22 ± 4%
Compound **3**	16 ± 4%
Hydroquinone	16 ± 1%
Kojic acid	23 ± 1%

**Table 2:** Cell viability of compound **1** in HEMs and HDFs. Results indicate mean ± SD (*n* = 3).

Treatment	Melanocytes		Fibroblasts	
	Cell Viability	Student’s *t*-Test	Cell Viability	Student’s *t*-Test
Control	100 ± 1%	-	100 ± 5%	-
3.125 µM	96 ± 4%	0.086	95 ± 11%	0.290
31.25 µM	101 ± 4%	0.333	78 ± 14%	0.134
156.25 µM	103 ± 7%	0.260	83 ± 16%	0.259
312.5 µM	89 ± 8%	0.094	83 ± 14%	0.111
1562.5 µM	49 ± 3%	0.003 **	60 ± 1%	0.035 *

* *p* < 0.05, ** *p* < 0.01.

## Data Availability

ATAC-Seq data are not shown due to privacy issues. The data presented in this study are available upon request to the corresponding author.

## Supplementary Materials

The following supporting information can be downloaded at: https://www.mdpi.com/article/10.3390/pharmaceutics16111405/s1, Table S1: List of primers (related to ATAC-Seq assay) used for qPCR assay; Table S2: List of autophagy primers used for qPCR assay; Table S3: List of miRNA primers used for qPCR assay; Figure S1: Synthetic scheme for compounds **1**, **2** and **3**; Figure S2: Infrared spectroscopy spectrum of compound **1** using a FTIR-4600 spectrometer (Jasco, Spain); Figure S3: Histological melanin semiquantification by Fontana-Masson image analysis of reconstructed human pigmented epidermis (phototype VI) treated for 8 days with PBS (Control); Figure S4: Histological melanin semiquantification by Fontana-Masson image analysis of reconstructed human pigmented epidermis (phototype VI) treated for 8 days with 0.5% *w*/*w* Compound **1**.

## Abbreviations

AGE: advanced glycation end product; ATAC-seq, assay for transposase-accessible chromatin using sequencing; BSA, bovine serum albumin; DMSO, dimethyl sulfoxide; GYA, glyceraldehyde; HDF, human dermal fibroblast; HEM, human epidermal melanocyte; HPLC, high-performance liquid chromatography; RHE, reconstructed human epidermis; RHPE, reconstructed human pigmented epidermis; RT, room temperature; SRB, sulforhodamine B.

## References

[1] Park H.Y., Kosmadaki M., Yaar M., Gilchrest B.A. (2009). Cellular mechanisms regulating human melanogenesis. Cell. Mol. Life Sci..

[2] Bastonini E., Kovacs D., Picardo M. (2016). Skin pigmentation and pigmentary disorders: Focus on epidermal/dermal cross-talk. Ann. Dermatol..

[3] Zolghadri S., Bahrami A., Hassan Khan M.T., Munoz-Munoz J., Garcia-Molina F., Garcia-Canovas F., Saboury A.A. (2019). A comprehensive review on tyrosinase inhibitors. J. Enzym. Inhib. Med. Chem..

[4] Masum M.N., Yamauchi K., Mitsunaga T. (2019). Tyrosinase inhibitors from natural and synthetic sources as skin-lightening agents. Rev. Agric. Sci..

[5] Nerya O., Musa R., Khatib S., Tamir S., Vaya J. (2004). Chalcones as potent tyrosinase inhibitors: The effect of hydroxyl positions and numbers. Phytochemistry.

[6] Kostopoulou I., Detsi A. (2018). Recent developments on tyrosinase inhibitors based on the chalcone and aurone scaffolds. Curr. Enzym. Inhib..

[7] Khatib S., Nerya O., Musa R., Shmuel M., Tamir S., Vaya J. (2005). Chalcones as potent tyrosinase inhibitors: The importance of a 2,4-substituted resorcinol moiety. Bioorganic Med. Chem..

[8] Gomes M.N., Muratov E.N., Pereira M., Peixoto J.C., Rosseto L.P., Cravo P.V.L., Andrade C.H., Neves B.J. (2017). Chalcone derivatives: Promising starting points for drug design. Molecules.

[9] Elkanzi N.A.A., Hrichi H., Alolayan R.A., Derafa W., Zahou F.M., Bakr R.B. (2022). Synthesis of Chalcones Derivatives and Their Biological Activities: A Review. ACS Omega.

[10] Rajendran G., Bhanu D., Aruchamy B., Ramani P., Pandurangan N., Bobba K.N., Oh E.J., Chung H.Y., Gangadaran P., Ahn B.-C. (2022). Chalcone: A Promising Bioactive Scaffold in Medicinal Chemistry. Pharmaceuticals.

[11] Shin S., Kum H., Ryu D., Kim M., Jung E., Park D. (2014). Protective Effects of a New Phloretin Derivative against UVB-Induced Damage in Skin Cell Model and Human Volunteers. Int. J. Mol. Sci..

[12] Qi Z., Liu M., Liu Y., Zhang M., Yang G. (2014). Tetramethoxychalcone, a Chalcone Derivative, Suppresses Proliferation, Blocks Cell Cycle Progression, and Induces Apoptosis of Human Ovarian Cancer Cells. PLoS ONE.

[13] Okolo E.N., Ugwu D.I., Ezema B.E., Ndefo J.C., Eze F.U., Ezema C.G., Ezugwu J.A., Ujam O.T. (2021). New chalcone derivatives as potential antimicrobial and antioxidant agents. Sci. Rep..

[14] Rubinsztein D.C., Mariño G., Kroemer G. (2011). Autophagy and aging. Cell.

[15] Chaudhuri J., Bains Y., Guha S., Kahn A., Hall D., Bose N., Gugliucci A., Kapahi P. (2018). The Role of Advanced Glycation End Products in Aging and Metabolic Diseases: Bridging Association and Causality. Cell Metab..

[16] Kammeyer A., Luiten R.M. (2015). Oxidation events and skin aging. Ageing Res. Rev..

[17] Rolt A., Cox L.S. (2020). Structural basis of the anti-ageing effects of polyphenolics: Mitigation of oxidative stress. BMC Chem..

[18] Katoh K., Rozewicki J., Yamada K.D. (2018). MAFFT online service: Multiple sequence alignment, interactive sequence choice and visualization. Brief. Bioinform..

[19] Webb B., Sali A. (2016). Comparative protein structure modeling using MODELLER. Curr. Protoc. Bioinform..

[20] Matoba Y., Kumagai T., Yamamoto A., Yoshitsu H., Sugiyama M. (2006). Crystallographic evidence that the dinuclear copper center of tyrosinase is flexible during catalysis. J. Biol. Chem..

[21] Holt P.A., Chaires J.B., Trent J.O. (2008). Molecular docking of intercalators and groove-binders to nucleic acids using Autodock and Surflex. J. Chem. Inf. Model..

[22] Vichai V., Kirtikara K. (2006). Sulforhodamine B colorimetric assay for cytotoxicity screening. Nat. Protoc..

[23] Buenrostro J.D., Giresi P.G., Zaba L.C., Chang H.Y., Greenleaf W.J. (2013). Transposition of native chromatin for fast and sensitive epigenomic profiling of open chromatin, DNA-binding proteins, and nucleosome position. Nat. Methods.

[24] Corces M.R., Trevino A.E., Hamilton E.G., Greenside P.G., Sinnott-Armstrong N.A., Vesuna S., Satpathy A.T., Rubin A.J., Montine K.S., Wu B. (2017). An improved ATAC-seq protocol reduces background and enables interrogation of frozen tissues. Nat. Methods.

[25] Li S., Zong X., Zhang L., Li L., Wu J. (2022). A chromatin accessibility landscape during early adipogenesis of human adipose-derived stem cells. Adipocyte.

[26] Winder A.J., Harris H. (1991). New assays for the tyrosine hydroxylase and dopa oxidase activities of tyrosinase. Eur. J. Biochem..

[27] Qiao Z., Koizumi Y., Zhang M., Natsui M., Flores M.J., Gao L., Yusa K., Koyota S., Sugiyama T. (2012). Anti-melanogenesis effect of *Glechoma hederacea* L. extract on B16 murine melanoma cells. Biosci. Biotechnol. Biochem..

[28] Liang C.C., Park A.Y., Guan J.L. (2007). In vitro scratch assay: A convenient and inexpensive method for analysis of cell migration in vitro. Nat. Protoc..

[29] Watanabe T., Hasegawa T., Takahashi H., Ishibashi T., Itagaki H., Sugibayashi K. (2002). Utility of MTT assay in three-dimensional cultured human skin model as an alternative for Draize skin irritation test: Approach using diffusion law of irritant in skin and toxicokinetics-toxicodynamics correlation. Pharm. Res..

[30] Huxtable R.J. (2013). Biochemistry of Sulfur.

[31] Lai X., Wichers H.J., Soler-Lopez M., Dijkstra B.W. (2017). Structure of Human Tyrosinase Related Protein 1 Reveals a Binuclear Zinc Active Site Important for Melanogenesis. Angew. Chem. Int. Ed..

[32] Slominski A., Zmijewski M.A., Pawelek J. (2012). L-tyrosine and L-dihydroxyphenylalanine as hormone-like regulators of melanocyte functions. Pigment Cell Melanoma Res..

[33] Narsinghani T., Sharma M.C., Bhargav S. (2013). Synthesis, docking studies and antioxidant activity of some chalcone and aurone derivatives. Med. Chem. Res..

[34] Hushcha Y., Blo I., Oton-Gonzalez L., Di Mauro G., Martini F., Tognon M., De Mattei M. (2021). MicroRNAs in the regulation of melanogenesis. Int. J. Mol. Sci..

[35] Brown T.J., Kollara A., Shathasivam P., Ringuette M.J. (2019). Ventricular Zone Expressed PH Domain Containing 1 (VEPH1): An adaptor protein capable of modulating multiple signaling transduction pathways during normal and pathological development. Cell Commun. Signal..

[36] Yoshida H., Okada Y. (2019). Role of HYBID (Hyaluronan binding protein involved in hyaluronan depolymerization), alias KIAA1199/CEMIP, in hyaluronan degradation in normal and photoaged skin. Int. J. Mol. Sci..

[37] Yoshida H., Aoki M., Komiya A., Endo Y., Kawabata K., Nakamura T., Sakai S., Sayo T., Okada Y., Takahashi Y. (2020). HYBID (alias KIAA1199/CEMIP) and hyaluronan synthase coordinately regulate hyaluronan metabolism in histamine-stimulated skin fibroblasts. J. Biol. Chem..

[38] Kim H.S., Park S.Y., Moon S.H., Lee J.D., Kim S. (2018). Autophagy in human skin fibroblasts: Impact of age. Int. J. Mol. Sci..

[39] Perrot G., Colin-Pierre C., Ramont L., Proult I., Garbar C., Bardey V., Jeanmaire C., Mine S., Danoux L., Berthélémy N. (2019). Decreased expression of GPC1 in human skin keratinocytes and epidermis during ageing. Exp. Gerontol..

[40] Applegate L.A., Scaletta C., Panizzon R. (1998). Evidence that ferritin is UV inducible in human skin: Part of a putative defense mechanism. J. Investig. Dermatol..

[41] Gruber J.V., Holtz R. (2013). Examining the impact of skin lighteners in vitro. Oxid. Med. Cell. Longev..

[42] Schallreuter K.U., Wood J.M. (2001). Thioredoxin Reductase—Its Role in Epidermal Redox Status. J. Photochem. Photobiol. B Biol..

[43] Cadenas C., Franckenstein D., Schmidt M., Gehrmann M., Hermes M., Geppert B., Schormann W., Maccoux L.J., Schug M., Schumann A. (2010). Role of thioredoxin reductase 1 and thioredoxin interacting protein in prognosis of breast cancer. Breast Cancer Res..

[44] Zeng Z., Sharma A., Ju L., Murai J., Umans L., Vermeire L., Pommier Y., Takeda S., Huylebroeck D., Caldecott K.W. (2012). TDP2 promotes repair of topoisomerase I-mediated DNA damage in the absence of TDP1. Nucleic Acids Res..

[45] Gilbert N., Ramsahoye B. (2005). The relationship between chromatin structure and transcriptional activity in mammalian genomes. Brief. Funct. Genom..

[46] Jones P.L., Wolffe A.P. (1999). Relationships between chromatin organization and DNA methylation in determining gene expression. Semin. Cancer Biol..

[47] Zaccaria M., Ludovici M., Sanzani S.M., Ippolito A., Cigliano R.A., Sanseverino W., Scarpari M., Scala V., Fanelli C., Reverberi M. (2015). Menadione-induced oxidative stress re-shapes the oxylipin profile of *Aspergillus flavus* and its lifestyle. Toxins.

[48] Nguyen H.P., Katta R. (2015). Sugar Sag: Glycation and the Role of Diet in Aging Skin. Ski. Ther. Lett..

[49] Alikhani Z., Alikhani M., Boyd C.M., Nagao K., Trackman P.C., Graves D.T. (2005). Advanced glycation end products enhance expression of pro-apoptotic genes and stimulate fibroblast apoptosis through cytoplasmic and mitochondrial pathways. J. Biol. Chem..

