# Supplementary material for: New Chalcone-Derived Molecule for the Topical Regulation of Hyperpigmentation and Skin Aging
Source: Pharmaceutics. 2024 Oct 31;16(11):1405. doi: 10.3390/pharmaceutics16111405 (PMC11597169; doi:10.3390/pharmaceutics16111405)

SUPPLEMENTARY MATERIAL

Supplementary Table S1. List of primers (related to ATAC-SEQ assay) used for qPCR assay.

Gene	Forward	Reverse
CEMIP	5′-TGCTGCCCGGGTATTCAAAT-3'	5’-CGTCCACTCCACGTCTTGAA-3′
PRPF6	5′-AAGCTGACTCCTGTTCCTGA-3’	5′-TCATTCCACCTGGGTATGGAG-3’
VEPH1	5′CAGAATATATCTGATGCCCACAAAA-3’	5′-TCATTCCACCTGGGTATGGAG-3’
TXNRD1	5′-CCACTGGTGAAAGACCACGTT-3′	5′-AGGAGAAAAGATCATCAC-TGCTGAT-3′
FTH1	5′-AAGCTGCAGAACCAACGAGG-3’	5′-AGTCACACAAATGGGGGTCATT-3’
TDP2	5′-CTGGGAGATGGAAAGGGCTC-3’	5′-AGAGATGGTTTCAGGTCGGC-3’
GPC1	5′-TGCCCTGACTATTGCCGAA-3’	5′-CATGGAGTCCAGGAGGTTCCT-3’
FN1	5’-GTGCCTGATGTGGCCTTTTC-3’	5’-GACTCACACACCTATGGGCT-3’

Supplementary Table S2. List of autophagy primers used for qPCR assay.

Gene	Forward	Reverse
BECN1	5’-AGCTGCCGTTATACTGTTCTG-3’	5’- CTGCCTCCTGTGTCTTCAATCTT-3’
LC3	5’-GATGTCCGACTTATTCGAGAGC-3’	5’-TTGAGCTGTAAGCGCCTTCTA-3’
p62	5′-CCAGCACCAAGAGCAC-GGACAGCG-3′	5′-TGGGGAGAAGAAGGGGACCACGAA-3′
VMP1	5’-ACTCTTTTGCTGGAAGCGGT-3’	5’-GGAACACTGGCAAACCAACT-3’
ATG12	5’-TAGAGCGAACACGAACCATCC-3’	5’-CACTGCCAAAACACTCATAGAGA-3’
ATG5	5’-GTTTTGGGCCATCAATCGGAA-3’	5’-TCTCCTAGTGTGTGCAACTGT-3’
ATG7	5’-ATGATCCCTGTAACTTAGCCCA-3’	5’-CACGGAAGCAAACAACTTCAAC-3’

Supplementary Table S3. List of miRNA primers used for qPCR assay.

**Gene**	**Forward**
miR-125	5'-ACGGGTTAGGCTCTTGGGAGCT-3'
miR-137	5'-TTATTGCTTAAGAATACGCGTAG-3'
miR-145	5'-GTCCAGTTTTCCCAGGAATCCCT-3'
miR-21	5'-TAGCTTATCAGACTGATGTTGA-3'
miR-218	5'-TTGTGCTTGATCTAACCATGT-3'
miR-675	5'-CTGTATGCCCTCACCGCTCA-3'
miR-203	5'-GTGAAATGTTTAGGACCACTAG-3'

Supplementary Figure S1. Synthetic scheme for compounds **1**, **2** and **3**.

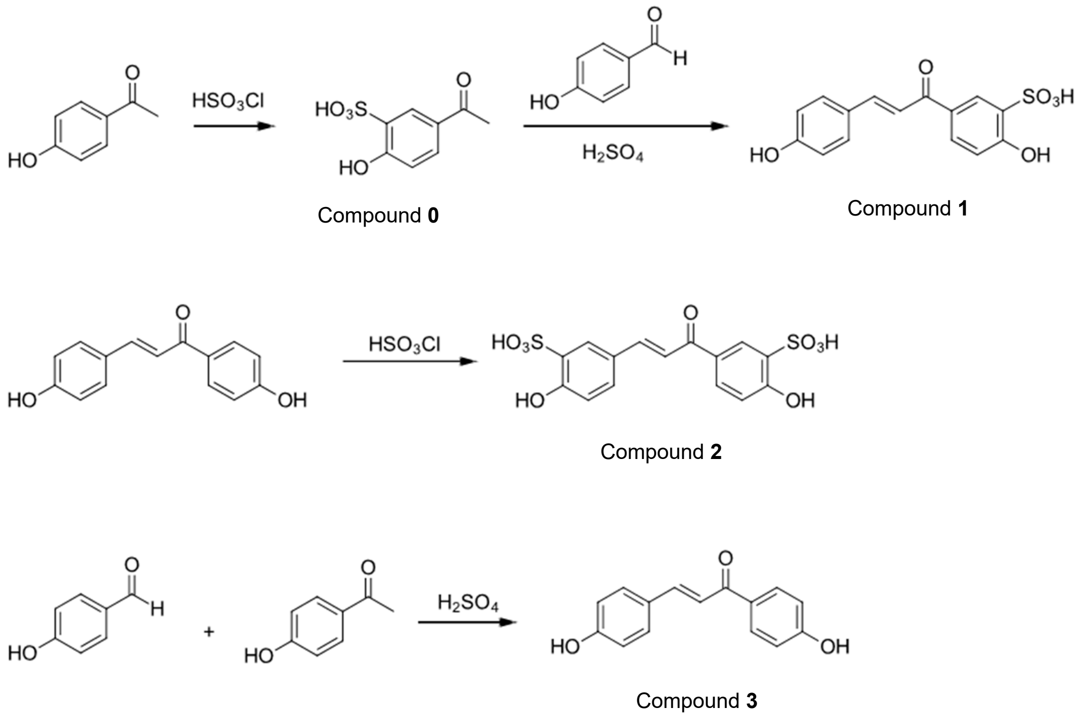

Supplementary Figure S2. Infrared spectroscopy spectrum of compound **1** using a FTIR-4600 spectrometer (Jasco, Spain).

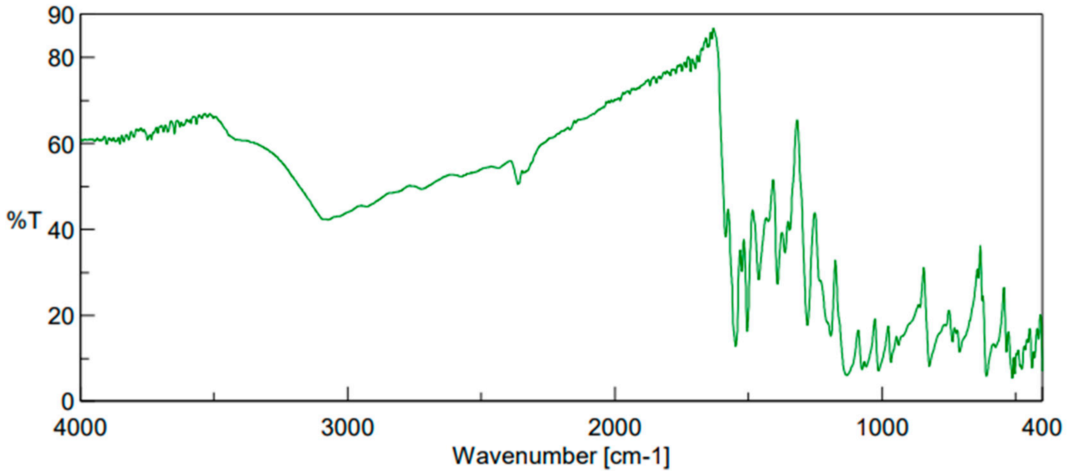

Supplementary Figure S3. Histological melanin semiquantification by Fontana-Masson image analysis of reconstructed human pigmented epidermis (phototype VI) treated for 8 days with PBS (Control).

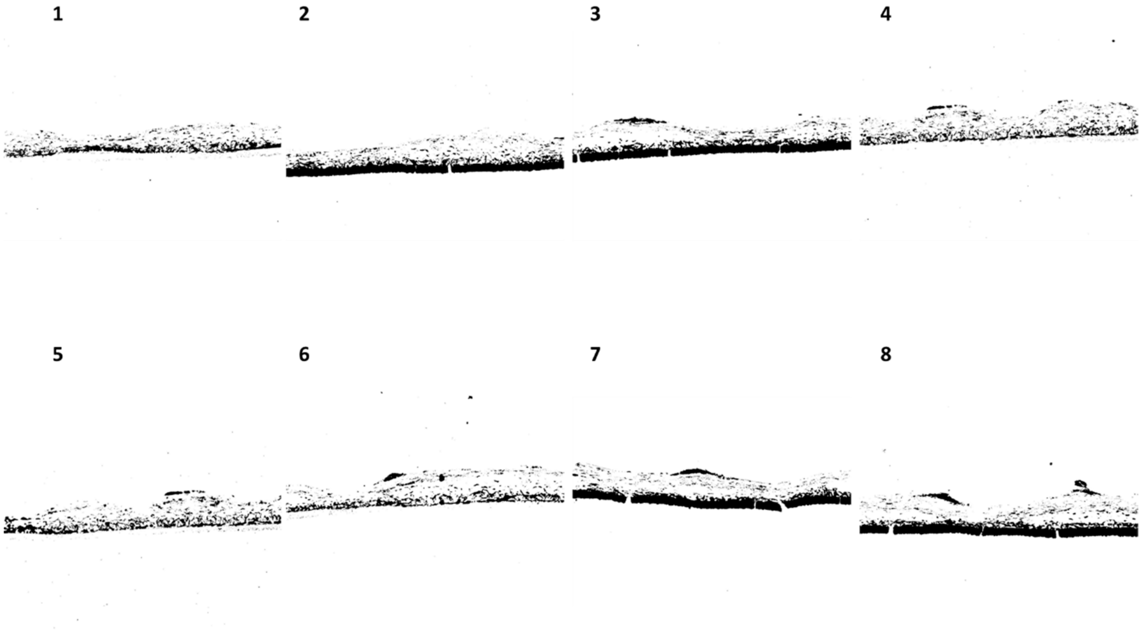

Supplementary Figure S4. Histological melanin semiquantification by Fontana-Masson image analysis of reconstructed human pigmented epidermis (phototype VI) treated for 8 days with 0.5% w/w Compound **1**.

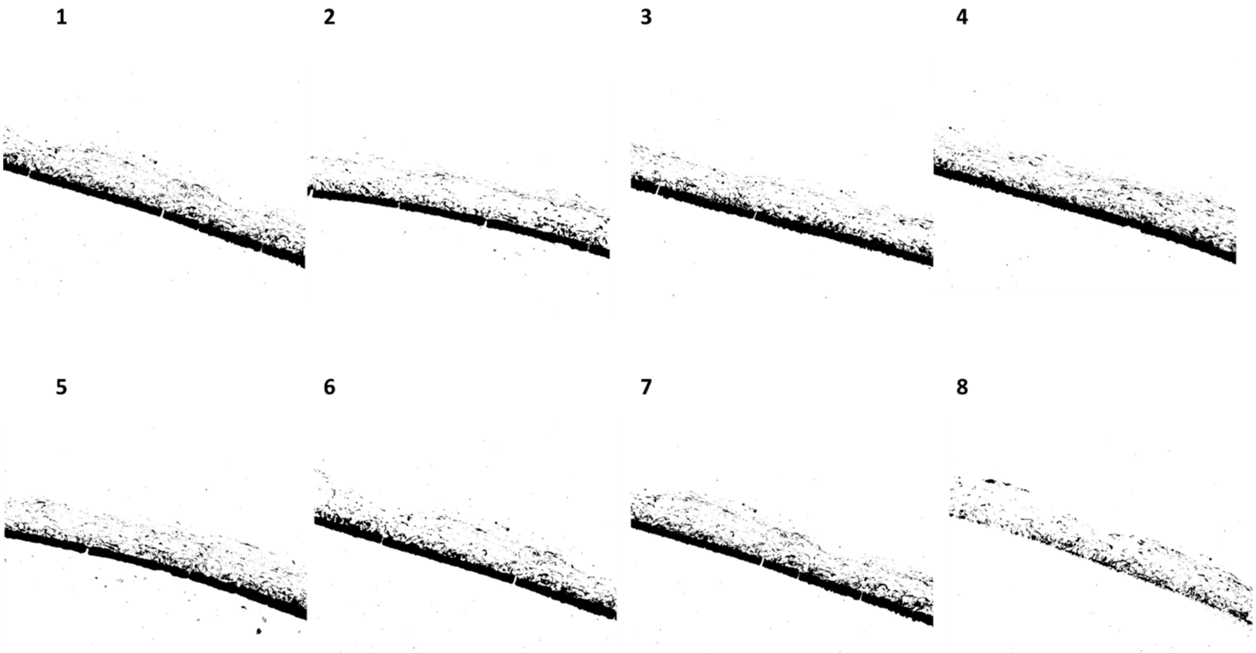

Supplement: Supplementary file 1 [file pharmaceutics-16-01405-s001.zip › pharmaceutics-3272818-supplementary.pdf]
